# Compound probiotics promote the growth of piglets through activating the JAK2/STAT5 signaling pathway

**DOI:** 10.3389/fmicb.2025.1480077

**Published:** 2025-05-13

**Authors:** Zhiyuan Li, Xueyan Dai, Fan Yang, Weina Zhao, Zhiwei Xiong, Wengen Wan, Guoyun Wu, Tianfang Xu, Huabin Cao

**Affiliations:** ^1^Jiangxi Provincial Key Laboratory for Animal Health, Institute of Animal Population Health, College of Animal Science and Technology, Jiangxi Agricultural University, Nanchang, China; ^2^Jiangxi Agricultural Technology Extension Center, Nanchang, China; ^3^Jiangxi Biotech Vocational College, Nanchang, Jiangxi, China

**Keywords:** compound probiotics, growth performance, JAK2/STAT5 pathway, liver, piglet

## Abstract

**Introduction:**

Composite probiotics are characterized by their non-resistance, no residues, non-toxic effects, and pollution-free nature. To investigate their application capability in piglets, this study aims to explore the effects of composite probiotics on the growth performance and related physiological indicators of piglets.

**Methods:**

Sixteen 30-day-old healthy weaned “Duroc × Landrace × Large White” piglets were randomly divided into a control group and a composite probiotic (CP) group. The CP group was supplemented with 2 mg·kg-1 of composite probiotics in the basal diet. After 120 days, the growth performance, serum biochemical parameters, hepatic hormones, and expression levels of JAK2/STAT5 signaling pathway-related factors were measured in each group.

**Results:**

The average daily gain of piglets in the CP group significantly increased, and the feed conversion ratio significantly decreased (*p* < 0.05). Among the serum biochemical parameters, the levels of triglyceride (TG), aspartate aminotransferase (AST), total cholesterol (TC), and alanine aminotransferase (ALT) were all elevated. Additionally, the levels of growth hormone, insulin, thyroid-stimulating hormone, and triiodothyronine also significantly increased. Furthermore, the addition of composite probiotics upregulated the expression levels of GHR, JAK2, STAT3, STAT5a, and STAT5b.

**Discussion:**

In conclusion, composite probiotics effectively promote the growth performance of piglets by activating the JAK2/STAT5 signaling pathway. These findings provide a theoretical basis for the application of composite probiotics in piglet feeding.

## Introduction

Over the past decades, antibiotics have served some functions extremely well, such as disease prevention and improvement of growth performance (Li et al., 2018). Recent projections suggested that a 67% increase consumption of antimicrobials in global, with estimates indicating that consumption will rise from 63,151 ± 1,560 tons to 105,596 ± 3,605 tons (Van Boeckel et al., 2015). Unfortunately, the indiscriminate use of antibiotics has been shown to potentially promote antibiotic-resistant bacteria while also disrupting the balance of beneficial bacteria. The World Health Organization (WHO) and Food and Agriculture Organization (FAO) have recommended that the use of moderate amounts of probiotics could improve animal health since 2001 (American Córdoba Park Hotel, Córdoba, Argentina, 2001). Multiple investigations have further demonstrated that probiotics could improve metabolic levels and growth performance in animals (Liu et al., 2023). Some studies indicated that the incorporation of bifidobacterium into feed could increase growth performance in broilers (Zhang et al., 2022). Additionally, a probiotic can also regulate intestinal microbiota, fostering a balanced symbiotic environment that enables the intestinal tract to withstand both infectious and non-infectious stressors (Shehata et al., 2022). It was determined that the addition of probiotics (*Lactobacillus*, *Clostridium butyricum*, *Enterococcus faecalis*, and *Bifidobacterium infantis*) to pigs’ diets could improve growth performance, feed conversion efficiency, average daily gain, growth rate, intestinal microflora regulation, nutrient utilization, intestinal health, and immune system regulation (Gareau et al., 2010; Cho et al., 2011; Dowarah et al., 2017; Wang et al., 2019). Meanwhile, a previous study has shown that supplementing with a variety of probiotic lactobacilli can improve liver function in pigs (Kim et al., 2021). The liver is a crucial parenchymal organ in pigs that governs various physiological processes, notably metabolism, feeding efficiency, and growth (Zhao et al., 2016; Xu et al., 2018; Horodyska et al., 2019). However, the role of probiotics in promoting animal growth via the modulation of energy metabolism in the liver remains unclear.

The Janus Kinase-signal Transducers and Activators of Transcription (JAK/STAT) signaling pathway plays a critical role in various physiological processes, including cell growth, differentiation, and immune response. It serves as an important downstream mediator for a variety of cytokines, hormones, and growth factors, such as the growth hormone receptor (GHR) and insulin-like growth factor 1 (IGF-1) that are the most important growth-related factors, mainly controlling the growth of skeletal muscle. Studies have shown that the JAK/STAT pathway could modulate the differentiation and metabolism of adipocytes, affect insulin (INS) signaling, and also participate in muscle metabolism (Himpe and Kooijman, 2009). Furthermore, the JAK2/STAT5 pathway could regulate whole-body energy metabolism by modulating the metabolic activity of the liver and intestine (Dodington et al., 2018). Other studies have demonstrated that the JAK2/STAT5 signaling pathway regulated bone development, homeostasis, and regrowth (Damerau et al., 2020; Sanpaolo et al., 2020), suggesting that enhanced bone growth could potentially boost skeletal muscle growth. However, few study examined whether probiotics affect energy metabolism through the JAK/STAT pathway.

*Rhodopseudomonas sphaeroides*, *Saccharomyces cerevisiae Hansen*, and *Bifidobacterium bifidum* are the important constituents of the compound probiotics. *Rhodopseudomonas sphaeroides* can withstand acid and bile salts, maintaining the homeostasis of gastrointestinal hormones (Zhou et al., 2007). *Saccharomyces cerevisiae* can improve piglet performance (Kiros et al., 2019). *Bifidobacterium* can synthesize vitamins B1, B2, B6, VK, and other vitamins, as well as a variety of amino acids. Therefore, the fermented products of *Bifidobacterium* can be used as a good source of B vitamins and some amino acids. Lactic acid and acetic acid produced by *Bifidobacterium* fermentation of carbohydrates that can reduce intestinal pH and facilitate the absorption of calcium, iron, and vitamin D. It can be speculated that adding a compound probiotic agent to the diet can improve the metabolic level of the body and promote the growth of animals. Therefore, it deserves further study whether a compound probiotic consisting of *Rhodopseudomonas sphaeroides*, *Saccharomyces cerevisiae Hansen*, and *Bifidobacterium* can promote pig growth through the JAK2/STAT5 signaling pathway.

## Materials and methods

### Ethical approval

The study was conducted according to the guidelines of Animal Management and Ethics Committee of Jiangxi Agricultural University (NO. JXAULL-2023-33).

### Source of compound probiotics microbial agent

Compound probiotics microbial agent was provided by Ruibote Biological Technology Co., Ltd. (Fuzhou, Jiangxi, China) in this study. *Rhodopseudomonas sphaeroides*, *Saccharomyces cerevisiae Hansen* and *Bifidobacterium bifidum* could be used at a ratio of 1:2:1.8, and the total number of viable bacteria >2.0 × 10^7^ CFU/g.

### Animals and treatment

The program of this study was approved by the ethics committee of the local institute. The following procedures were approved by The Animal Management and Ethics Committee of the Jiangxi Agricultural University (Nanchang, China). Sixteen healthy 30-days old Duroc × Yorkshire × Landrace weaned piglets were randomly and equally divided into two groups: control group (feeding a basal diet) and CP group (feeding compound probiotics microbial inoculant fermented feed with 2 g/Kg basal diet compound probiotics). Meal basal diet (Table 1) was formulated based on National Research Council 2012 (NRC 2012). The compound probiotics microbial agent fermented feed was obtained after 24 h incubation at room temperature. During the experimental period, deworming and routine immunization of the weaned piglets were conducted in accordance with the management system of the original pig farm, allowing all pigs free access to food. The pigs’ body weights were measured regularly, and their daily feed intake was recorded. After 120 days of the experiment, the experimental pigs were fasted for 12 h. Following intravenous injection of an adequate dose of pentobarbital sodium to induce anesthesia, experimental samples were collected. No adverse events occurred throughout the entire experimental process.

**Table 1:** Composition and nutrient levels of the basal diet (air-drybasis) %.

**Ingredients**	**Content (%)**
Soybean oil	1.65
Soybean	16.30
Soybean meal	7.00
Intestinal membrane protein powder	2.50
Fish meal	6.25
Whey powder	55.80
Corn	4.50
Bran	0.25
Lysine	0.10
Methionine	1.05
Stone powder	0.80
CaHPH4	0.30
NaCl	1.00
Total	100

**Nutrient level (g/Kg)**	**Content (%)**
Total phosphorus	0.60
Crude protein	19.63
Lys	1.32
Thr	0.81
Met + Cys	0.77
Ca	0.96
Dry matter	89.21
Met	0.43
Digestible energy (MJ·Kg^−1^)	14.36

The premixes provided Vit A 8000 IU, Vit D 2500 IU, Vit E 15 mg, Vit B 24.00 mg, Vit B1 2.00 mg, Vit B12 0.02 mg, biotin 0.06 mg, choline 0.5 g, Folic acid 0.2 g. Niacin 20 mg, pantothenic acid 10 mg, iron 110 mg, copper 165 mg. Manganese 80 mg, zinc 330 mg, selenium 0.2 mg.

### Growth performance

On the 30th, 60th, 90th, and 120th days of the experiment, the amount of feed consumed and body weight were measured. The average daily weight gain (ADG), average daily feed intake (ADFI), and feed conversion rate (FCR) were subsequently calculated.

### Sampling and sample processing

On the 120th day of the experiment, after a 12-h fasting period, blood samples (10 mL per pig) were collected from the anterior vena cava and transferred into 20 mL centrifuge tubes. The tubes were incubated at 37°C for 0.5 h in a constant-temperature chamber and then centrifuged at 3,500 g for 15 min. The serum from the upper layer was aspirated and stored at −80°C for subsequent analysis. Subsequently, the pigs were euthanized by overdosing with pentobarbital, and samples of liver tissue and longest dorsal muscle were collected. The fresh liver tissue was stored in a −80°C refrigerator for further experiments.

### Carcass performance

On the 120th day of the experiment, the carcass straight length, carcass oblique length, back fat and skin thickness of pigs were measured according to the standard DB43/T356-2007 (China).

### Determined the serum biochemical indices

After 120 days of treatment, blood samples were drawn from the jugular vein. The serum was collected from the top layer after centrifugation. The concentrations of triglycerides (TG), total cholesterol (TC), alanine aminotransferase (ALT), and aspartate aminotransferase (AST) in the serum were measured using a Hitachi 3100 serum biochemical analyzer from Japan.

### Muscle flavor

A section of the longissimus dorsi muscle, with a specific thickness (approximately 2.5 cm), was cut at the level of the 10th rib. The meat color was assessed using a color chart under sufficient natural indoor lighting conditions. Fatty acids and amino acids were extracted from the longissimus dorsi muscle in accordance with the Chinese standard GB5009.168-2016, and analyzed using an Agilent 7890B gas chromatography instrument from the United States.

### Hormone level was measured

To obtain the supernatant of liver tissue, the manufacturer’s protocol was strictly followed. Quantitative assessment of thyroid stimulating hormone (TSH), growth hormone (GH), insulin (INS), and triiodothyronine (T3) levels in the liver tissue supernatant was conducted using ELISA on a precision microplate spectrophotometer. All ELISA kits were sourced from Nanjing Jiancheng, China.

### Real-time quantitative polymerase chain reaction

Following previous methods established in our laboratory (Li et al., 2018), total RNA was extracted from liver tissues by using Transzol UP reagent (TaKaRa Biotech, China) according to the manufacturer’s instructions. Subsequently, the extracted RNA was reverse-transcribed into complementary DNA (cDNA) using the EasyScript One-Step gDNA Removal and cDNA Synthesis SuperMix (TransGen, China) following the recommended protocol. Real-time PCR amplification of each sample was carried out using the QuantStudio7 Flex system, and the relative abundance of mRNA was determined using the 2^−ΔΔCT^ method, with normalization against the reference gene GAPDH. The Table 2 showed the design results of primer sequence.

**Table 2:** Primer sequences (F: Forward, R: Reverse).

Gene	5′-Primer (F)	3′-Primer (R)
GHR	TCCGTCACGGTTTACAGAGC	GGTGGATCCGGTTGCACTAT
JAK2	ATGTGAGTAGGAGCCGAACC	CTTCCGAAACCCGACGCT
STAT3	CCCCGTGTCTAATAGGGGAGA	ATCAGGGGTCACAACTGCTG
STAT5a	CAGCTAAAGCAGTGGACGGA	GAGAGTGAGCCCCTGGTAGA
STAT5b	GTGGTTTGATGGCGTGATGG	CTGGTCCATGTAGGTGGCATT
GAPDH	GAAGGTCGGTGTGAACGGAT	CCCATTTGATGTTAGCGGGAT

### Western blot

0.1 g of liver tissue was homogenized with 1 mL of RIPA and PMSF mixture (RIPA: PMSF = 100:1) in a 1.5 mL centrifuge tube at low temperature. The mixture was then incubated on ice and lysed for 30 min with intermittent mixing every 10 min. The centrifuge tube was then placed in a centrifuge at 4°C and 12,000 rpm for 10 min, and the supernatant was transferred to a new centrifuge tube. The obtained supernatant was measured using a BCA kit (Solarbio, Beijing, China) to determine the protein concentration. The sample was mixed with 6× loading buffer at a ratio of 1:5 and heat-treated in boiling water for 10 min. Protein electrophoresis, membrane transfer, sealing, antibody incubation, and color rendering were carried out. Finally, the gray value of the protein bands was analyzed using Image J (Version 1.54 m, Wayne Rasband and contributors, United States) software.

### Statistical analysis

Statistical analysis was conducted using Microsoft Excel 2019 (United States) and IBM SPSS 23.0 (United States). Bartlett’s test for homogeneity of variance and the Kolmogorov–Smirnov test for normality were applied to the measurement data, confirming that the data met the assumptions of homogeneity of variance and approximately followed a normal distribution. Independent sample *t*-tests were used to compare the two groups. All tests were two-tailed with α = 0.05. Data are presented as mean ± standard deviation and visualized as bar graphs using GraphPad Prism 8.0.1 software. Statistical significance was determined by the *p*-value, with * representing *p* < 0.05, ** representing *p* < 0.01, and *** representing *p* < 0.001.

## Results

### Compound probiotics could enhanced feeding piglets growth

**Figure 1:**
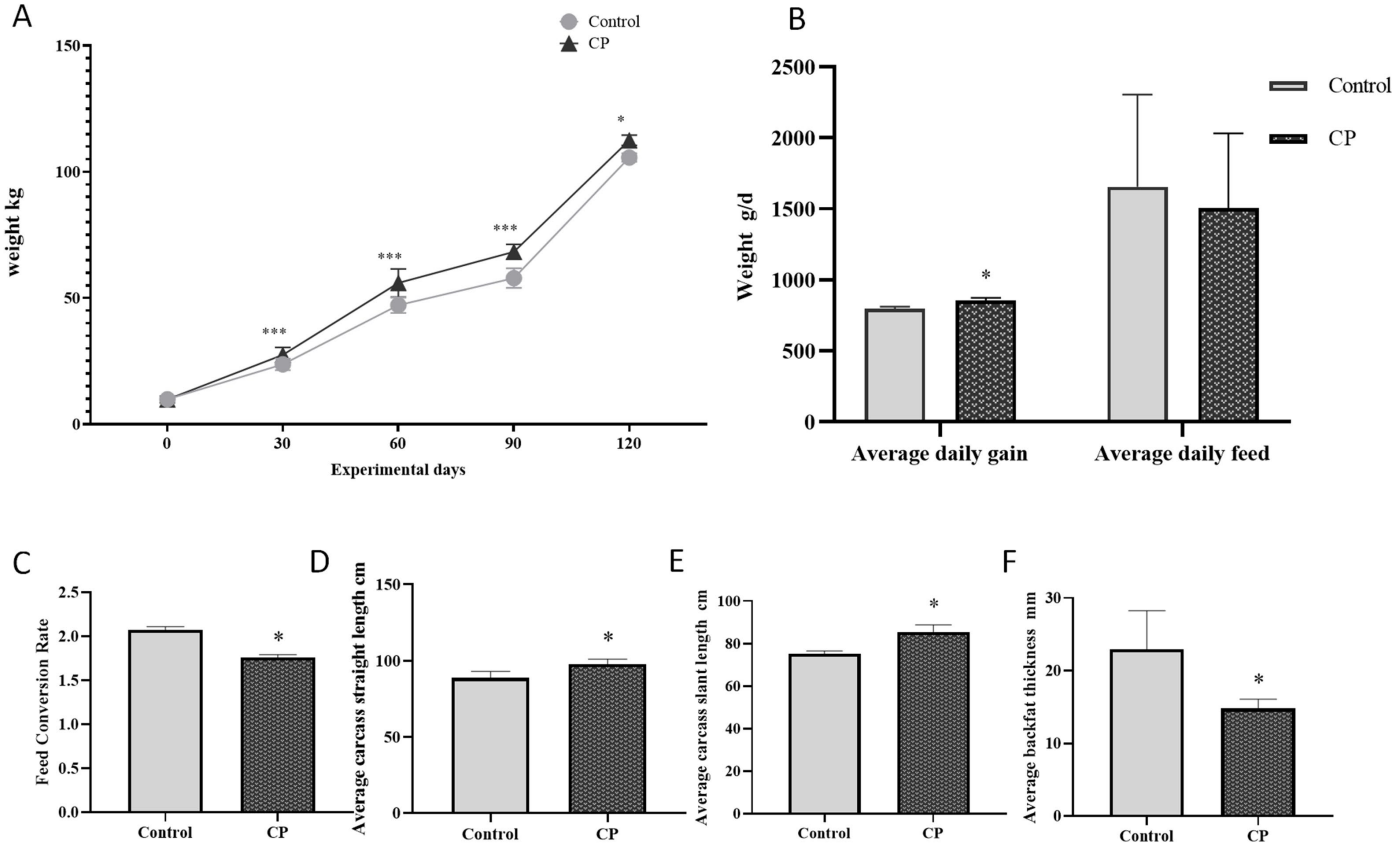
Compound probiotics could enhanced feeding piglets growth. **(A)** The weight curve; **(B)** The average daily gain and average daily feed; **(C)** Feed conversion rate (Figures 1A–C showed that in terms of initial body weight, average daily feed intake, and feed/gain ratio, there were no significant differences between the two groups). **(D)** Average carcass straight length; **(E)** Average carcass slant length; **(F)** Average backfat thickness. (However, the final body weight and average daily gain were significantly higher in the CP group than in the control group (*p* < 0.05). Additionally, the average carcass straight length and average carcass skew length had a significantly increased in the CP group, while the average back fat thickness was significantly decreased compared with the control group). “*” indicates a significant difference compared with the control group (*p < 0.05, **p < 0.01 and ***p < 0.001).

The results presented in Figures 1A–C showed that in terms of initial body weight, average daily feed intake, and feed/gain ratio, there were no significant differences between the two groups (*p* > 0.05). However, the final body weight and average daily gain were significantly higher in the CP group than in the control group (*p* < 0.05). Additionally, the average carcass straight length and average carcass skew length had a significantly increased in the CP group, while the average back fat thickness was significantly decreased compared with the control group (*p* < 0.05) (Figures 1D–F). These findings suggested that the addition of compound probiotics microbial agent fermented feed enhanced feeding piglets growth.

### Composite probiotics have a protective effect on piglet liver function

**Figure 2:**
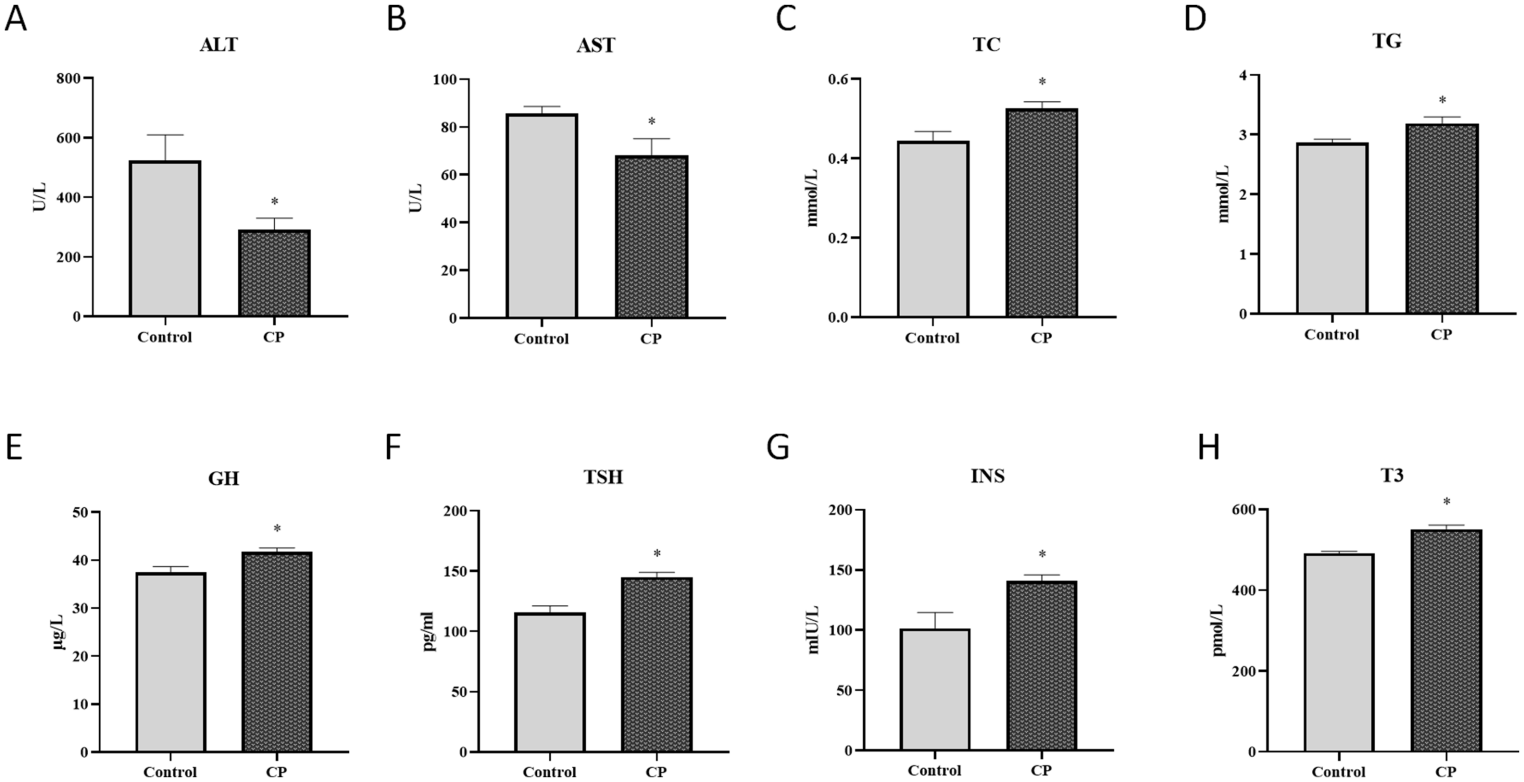
Composite probiotics have a protective effect on piglet liver function. **(A–D)** The serum biochemical indices levels [**A–D**, compared with the control group, the ALT and AST levels were significantly decreased in the CP group in serum (*p* < 0.05), but the TC and TG levels were significantly decreased]. **(E–H)** The liver hormone levels (**E–H** showed that the levels of GH, TSH, INS, and T3 were significantly increased in the CP group). “*” indicates a significant difference compared with the control group (*p < 0.05).

As shown in Figures 2A–D, compared with the control group, the ALT and AST levels were significantly decreased in the CP group in serum (*p* < 0.05), but the TC and TG levels were significantly decreased (*p* < 0.05). Figures 2E–H showed that the levels of GH, TSH, INS, and T3 were significantly increased in the CP group (*p* < 0.05).

### Composite probiotics increase the levels of amino acids and fatty acids in piglets

**Figure 3:**
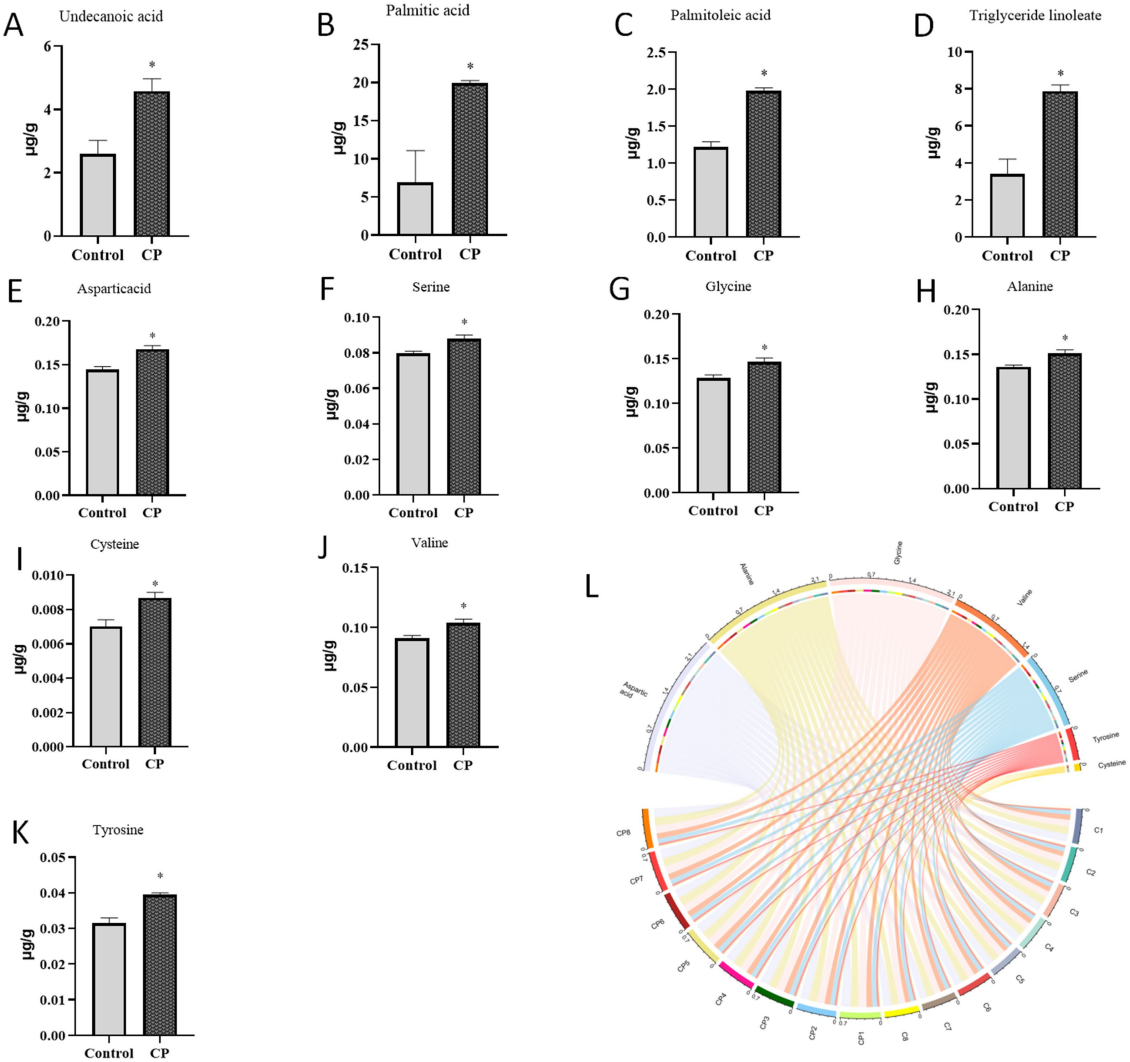
Composite probiotics increase the levels of amino acids and fatty acids in piglets. **(A–D)** The content of fatty acids (**A–D**, compared with the control group, the contents of carbamic acid, hexadecanoic acid, octadecanoic acid, erucic acid, and arachidonic acid were increased in the CP group). **(E–K)** The content of amino acids (**E–K**, the addition of compound probiotics could significantly increase the contents of aspartic acid, serine, glycine, alanine, cysteine, valine, and tyrosine). **(L)** The chord diagram of fatty acids and amino acids levels of control group and CP group in longissimus dorsi of pig. “*” indicates a significant difference compared with the control group (*p < 0.05).

As described in Figures 3A–D, compared with the control group, the contents of carbamic acid, hexadecanoic acid, octadecanoic acid, erucic acid, and arachidonic acid were increased in the CP group (*p* < 0.05). As shown in Figures 3E–K, the addition of compound probiotics could significantly increase the contents of aspartic acid, serine, glycine, alanine, cysteine, valine, and tyrosine (*p* < 0.05). Analyze the fatty acid and amino acid levels in the longissimus dorsi muscle of pigs in both the control group and the CP group using proportional chord diagrams Figure 3L.

### Composite probiotics can activate the JAK2/STAT5 signaling pathway in piglets

**Figure 4:**
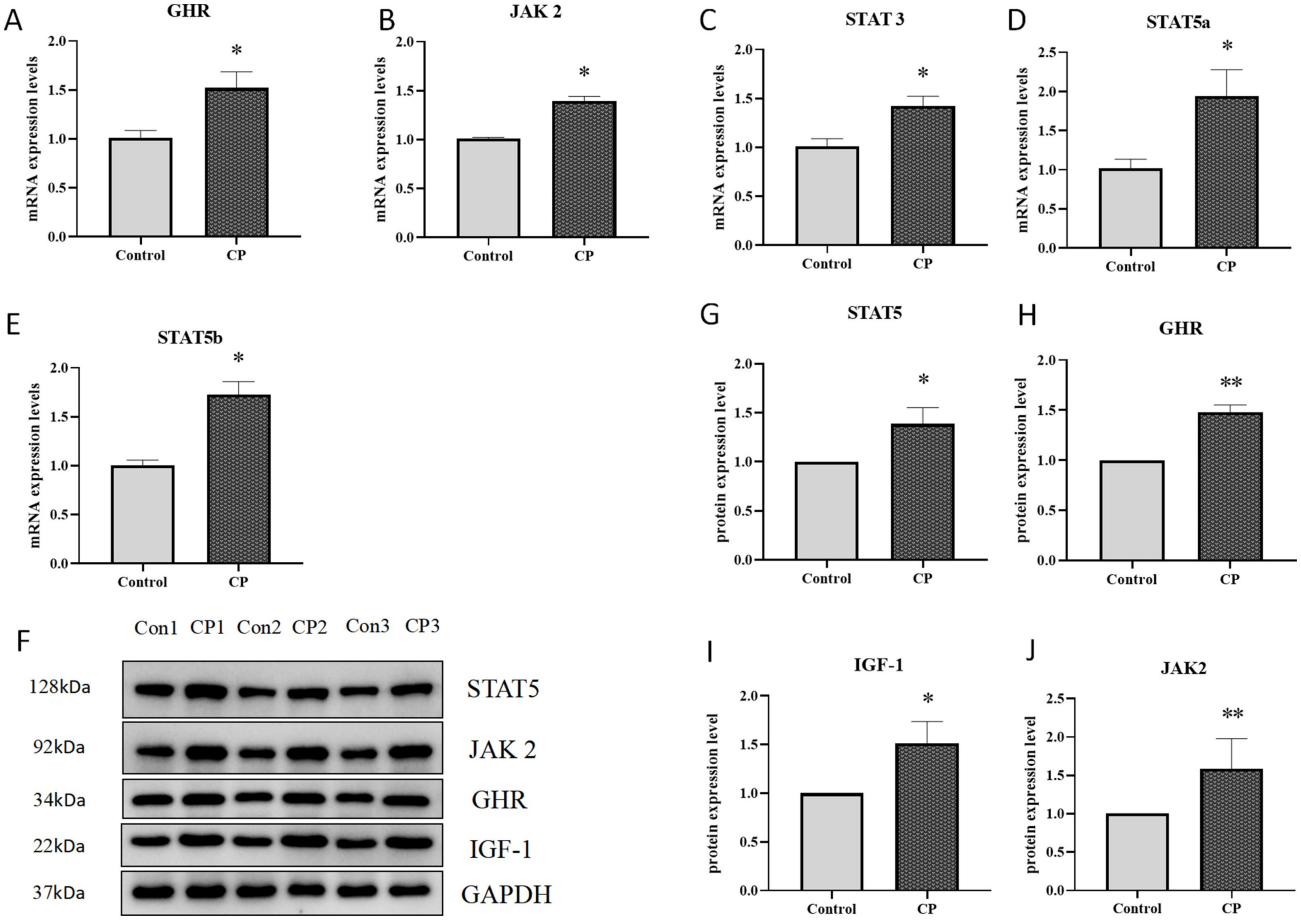
Composite probiotics can activate the JAK2/STAT5 signaling pathway in piglets. **(A–E)** The mRNA expression level of related factors in the JAK2/STAT5 signaling pathway (**A–E**, compared with the control group, the mRNA levels of Stat3, JAK2, GHR, Stat5b, and Stat5a genes were significantly upregulated in the CP group). **(F–J)** The protein expression level of related factors in the JAK2/STAT5 signaling pathway (**F–J**, the protein expression levels of STAT5, JAK2, GHR and IGF-1 has a dramatically increased). “*” indicates a significant difference compared with the control group (*p < 0.05, **p < 0.01).

As shown in Figures 4A–E, compared with the control group, the mRNA levels of Stat3, JAK2, GHR, Stat5b, and Stat5a genes were significantly upregulated in the CP group (*p* < 0.05). Similarly, the protein expression levels of STAT5, JAK2, GHR and IGF-1 has a dramatically increased (Figures 4F–J). These results indicated that compound probiotics activated the JAK2/STAT5 signaling pathway.

## Discussion

Animal growth and development is influenced by various factors, such as gene expression, endocrine, nutrient levels and environmental factors. Numerous investigations have validated that the ability of probiotics enhanced the animal growth performance (Liu et al., 2019; Saldana et al., 2019). Relevant literature indicates that in piglet breeding, the supplementation of probiotics can significantly improve their growth performance, enhance feed conversion rate, and reduce the incidence of diseases (Hansen et al., 2022; Xu et al., 2023). Abdel-Latif et al. reported that supplementary *C. butyricum* and *Saccharomyces cerevisiae* at an equal ratio in broilers feed significantly improved growth performance (Abdel-Latif et al., 2018). In the present study, the results also demonstrated that the addition of compound probiotics could enhance the average daily gain, decrease average daily feed, increased the contents of amino acids and fatty acids, activate the JAK2/STAT5 signaling pathway, and ultimately promoted growth performance.

Serum ALT and AST are important enzymes in the body, with their highest activities found in hepatocytes and cardiomyocytes, respectively. Under normal circumstances, the activities of glutamic-pyruvate transaminase and glutamic-oxalacetic transaminase in serum are relatively low. Increase of the permeability of the cell membrane of liver cells will cause a large amount of ALT and AST penetrate into the blood, result in increasing the activities of ALT and AST in the blood (Plaza-Diaz et al., 2014). A previous study has shown that supplementing with various strains of probiotic lactobacilli could improve pig liver function (Kim et al., 2021). Decreases both the content of AST and ALT in the CP group suggested that compound probiotics fermented feed has protective effect of the liver. Changes in TG and TC levels do not directly equate to changes in unsaturated fatty acid content, but as important indicators of lipid metabolism, alterations in their levels may indirectly reflect the metabolic status of unsaturated fatty acids in the body. Relevant literature suggests that elevated TG and TC levels provide energy and raw materials for the synthesis of unsaturated fatty acids, while also enhancing the activity of related synthetic enzymes. Our research results indicate that an increase in TG and TC levels suggests that composite probiotics could enhance the nutritional absorption of growing-finishing pigs, thereby promoting the synthesis of important substances such as unsaturated fatty acids (Hou et al., 2021; Shen et al., 2021). The liver is an important endocrine organ that synthesizes and secretes functional proteins like insulin-like growth factor 1. GH is the main endocrine hormone that regulates IGF-1, which mediates feeding, growth, development, metabolism, reproduction, and immune functions. INS, thyrotropin, and triiodothyronine are important hormones that affect the growth and development of pigs by promoting protein synthesis, stimulating cell proliferation and differentiation. Studies have shown that INS could promote protein synthesis, stimulate the proliferation and differentiation of various cells, the growth of connective tissue and bone marrow, and promote the growth of systemic organs (Jin Chan and Steiner, 2000; Bailes and Soloviev, 2021). INS can inhibit the enzyme of preadipocytes induced by INS, make lipid accumulation parallel to the change of enzyme activity, improve the level of soluble protein and glycerol phosphor dehydrogenase, and promote the differentiation of preadipocytes, which has been proved by Bluher et al. (2005). T3, secreted by thyroid, is an important hormone necessary for normal physiological functions of animals, and it plays an important role in material metabolism and energy metabolism, growth and development. T3 secretion is regulated by TSH. Moreover, previous research has shown that the content of GH and T3 increase significant elevated the growth and development (Miller et al., 1987; Bargi-Souza et al., 2017). In this study, GH, TSH, INS, and T3 levels were significantly elevated in the CP group, suggesting that combination probiotics could protect and promote the growth and development of piglets.

The amino acid composition of meat plays a fundamental role in determining protein nutrition and exerts a significant influence on meat quality (Erkkila et al., 2008). In parallel, fatty acids are essential nutrients and are limiting factors for the metabolism, growth, and reproduction of animals (Zhuang et al., 2018). Due to the body’s inability to synthesize certain essential fatty acids, such as linoleic acid and linolenic acid, their acquisition through dietary sources becomes imperative path (Leggio et al., 2012). The flavor of muscle is directly influenced by the composition and proportion of amino acids and fatty acids present in the muscle tissue, thereby serving as crucial indicators of meat flavor. Research has provided compelling evidence regarding the substantial influence of various factors on the meat flavor of ruminant animals, including the pre-slaughter feed proportion, the animals’ living environment, and the content and proportion of different substances in the meat after slaughter, with particular emphasis on fatty acids. Existing research has shown that the probiotic *Lactobacillus reuteri* 1 improves pork quality by increasing inosine monophosphate and glutamic acid (which may enhance flavor) and altering muscle fiber characteristics (Tian et al., 2021). Notably, glutamic acid, arginine, alanine, and glycine emerge as principal umami amino acids prominently present in muscle tissue, where their relative concentrations significantly shape the distinct flavor profile exhibited by meat. The result showed that compound probiotics could improve the umami taste of meat by increasing the amino acid content (amino acids, including aspartic acid, serine, glycine, alanine, cysteine, valine, and tyrosine), and enhance the nutritional value of meat by increasing the fatty acid content, which product high-quality meat.

The JAK/STAT signaling pathway is a common pathway for many cytokines and growth factors that regulate various cellular processes. JAK2 is a non-receptor tyrosine-protein kinase that plays an important role in signal transduction by inducing a cascade of cytoplasmic signals to regulate different cellular processes (Hackett et al., 1997). STAT5 are important members of the STATs family. It has two highly homologous subtypes STAT5a and STAT5b. STAT5 plays an important role in a wide range of physiological regulation of cell proliferation, differentiation, survival, and apoptosis (Varco-Merth and Rotwein, 2014). GHR is a transmembrane receptor protein that mediates the actions of GH by binding to it and initiating signal transduction pathways in cells (Qiu et al., 2017). Recently, research has shown that the expression of GHR could be modulated by the ingestion of postbiotics, which could have implications for enhancing its biological effects (Kareem et al., 2016; Izuddin et al., 2019). Numerous studies have demonstrated that JAK/STAT signaling pathway, as a downstream pathway of GHR, could regulate the synthesis and secretion of IGF-1, and thereby regulating the growth and development of pigs (Reindl et al., 2011; Chaudhari et al., 2017). IGFs mainly comprises IGF, IGFR, and IGF binding proteins (IGFBPs), which play a significant role in the growth, development, and metabolism of the organism. Among them, IGF-1 is an important endocrine factor of the animal growth axis, which plays vital roles in regulating the growth and development of the body and metabolism. Our study findings revealed that the supplementation of compound probiotics effectively enhanced animal growth through the modulation of key molecular players of the JAK/STAT signaling pathway, including JAK2, STAT5, GH, GHR, and IGF-1. A previous study has shown that *Clostridium butyricum*, as a probiotic, positively promotes the growth performance of weaned piglets, significantly enhancing their growth rate and overall health status (Cao et al., 2019). Our study findings revealed that the supplementation of compound probiotics effectively enhanced animal growth through the modulation of key molecular players of the JAK/STAT signaling pathway, including JAK2, STAT5, GH, GHR, and IGF-1. These findings underscore the potential of natural supplements to modulate key signaling pathways involved in animal growth and development.

## Conclusion

It is concluded that compound probiotics exhibit a beneficial effect on enhancing the growth performance of feeding piglets. Specifically, the CP group demonstrated significantly higher final body weight, average daily gain, carcass straight length, and carcass skew length, along with reduced back fat thickness compared to the control group. Furthermore, composite probiotics were found to protect piglet liver function by lowering ALT, AST, TC, and TG levels in serum while increasing GH, TSH, INS, and T3 levels. Additionally, the inclusion of compound probiotics increased the levels of various amino acids and fatty acids in piglets, contributing to improved nutritional status. Notably, compound probiotics activated the JAK2/STAT5 signaling pathway, as evidenced by upregulated mRNA and protein expression levels of related genes. These findings collectively suggest that compound probiotics hold great potential in promoting piglet growth and health, making them a valuable addition to piglet feeding strategies.

## Data Availability

The datasets presented in this study can be found in online repositories. The names of the repository/repositories and accession number(s) can be found in the article/supplementary material.
